# Effect of graphene oxide ratio on the cell adhesion and growth behavior on a graphene oxide-coated silicon substrate

**DOI:** 10.1038/srep33835

**Published:** 2016-09-22

**Authors:** Jin-Tak Jeong, Mun-Ki Choi, Yumin Sim, Jung-Taek Lim, Gil-Sung Kim, Maeng-Je Seong, Jung-Hwan Hyung, Keun Soo Kim, Ahmad Umar, Sang-Kwon Lee

**Affiliations:** 1 Department of Physics, Chung-Ang University, Seoul 156-756, Republic of Korea; 2 Department of Semiconductor Science and Technology, Chonbuk National University, Jeonju 561-756, Republic of Korea; 3 Department of Physics and Graphene Research Institute, Sejong University, Seoul 143-747, South Korea; 4 Department of Chemistry, College of Science and Arts, Najran University, Najran-11001, Kingdom of Saudi Arabia; 5 Promising Centre for Sensors and Electronic Devices (PCSED), Najran University, Najran-11001, Kingdom of Saudi Arabia

## Abstract

Control of living cells on biocompatible materials or on modified substrates is important for the development of bio-applications, including biosensors and implant biomaterials. The topography and hydrophobicity of substrates highly affect cell adhesion, growth, and cell growth kinetics, which is of great importance in bio-applications. Herein, we investigate the adhesion, growth, and morphology of cultured breast cancer cells on a silicon substrate, on which graphene oxides (GO) was partially formed. By minimizing the size and amount of the GO-containing solution and the further annealing process, GO-coated Si samples were prepared which partially covered the Si substrates. The coverage of GO on Si samples decreases upon annealing. The behaviors of cells cultured on two samples have been observed, i.e. partially GO-coated Si (P-GO) and annealed partially GO-coated Si (Annealed p-GO), with a different coverage of GO. Indeed, the spreading area covered by the cells and the number of cells for a given culture period in the incubator were highly dependent on the hydrophobicity and the presence of oxygenated groups on GO and Si substrates, suggesting hydrophobicity-driven cell growth. Thus, the presented method can be used to control the cell growth via an appropriate surface modification.

The control of living cells on functionalized substrates can lead to improved efficiencies in biosensors^1^,^2^ and biochips^3^,^4^. For example, isolated platforms for detecting circulating tumor cells are an important application of nanoscale devices in the biomedical environment. Nanostructures such as nanowires, nanopillars, and nanoholes are known to improve cell-capture efficiency by ~20% as reported previously^5^,^6^,^7^. This improvement is mainly due to enhanced cell adhesion to nanostructures with higher cell adhesion forces on the surface^6^,^8^,^9^,^10^,^11^. In addition, cell morphology on nanostructured surfaces can be characterized by the number of filopodia and their morphology^6^,^9^. Thus, surface topography, including aspect ratio of the surface and surface roughness, affects cell adhesion characteristics and, hence, is crucial for controlling the growth of living cells on substrates with various hydrophobicities.

Recently, the 2-dimensional (2D) carbon-based nanomaterials including graphene (Gr) and graphene oxide (GO) have been proposed as potential templates for studying the interactions between living cells and their coated or patterned surfaces^12^,^13^,^14^,^15^,^16^. For instance, Li and co-workers reported that the Gr substrates exhibited excellent biocompatibility and significantly promoted neurite sprouting and outgrowth of mouse hippocampal cells^15^. In case of tissue engineering field, many research groups are studying the stem cell differentiation using micro-patterned or Au-nanodotted GO surfaces^13^,^16^. These current issues have motivated us to study the effect of hydrophobicity and surface topography on the adhesion and growth behaviors of living cells using partially GO-coated samples. The GO is a two-dimensional material composed of an *sp*^2^ bonded carbon network, functionalized with hydroxyl, carbonyl, carboxyl, and carboxylate groups^17^,^18^,^19^. The properties of GO as soft membranes with high in-plane stiffness and high surface energy through bonded oxygen groups have many advantages for various applications, including biomedical applications, especially for cell growth behavior. The bonded hydroxyl, carbonyl, carboxyl, and carboxylate groups present on the graphene surface enable increased interaction with proteins through electrostatic, covalent, and hydrogen bonding. Hence, the increased proteins in the GO membrane strongly improve cell adhesion of cells attaching on the GO surface^20^,^21^,^22^. In addition, the GO possesses properties such as amphiphilicity, surface enhanced Raman scattering (SERS), and surface functionalization, and has been studied in many fields in addition to biological sensors for gene or drug delivery^23^,^24^. In this regard, the GO not only allows control of cell adhesion but also allows post-experimental analysis of cells and the drug response^23^,^24^. However, current research is focused on cell behavior and control on surfaces with GO and reduced GO, while not enough research has been performed on this topic in the case of partially covered GO surfaces, which has therefore been investigated in the current study.

In this study, we report cell adhesion and growth behavior on partially GO-coated (P-GO) and annealed partially GO-coated (Annealed p-GO) Si samples that are partially covered by GO films on Si substrates, as a function of incubation time extending up to 48 h. The samples were prepared by minimizing the size and amount of GO-containing solution and the further annealing process. Our experiments revealed that the most important factors influencing living cell behavior, including cell adhesion, growth, and morphology on GO-coated Si samples, include the coverage of GO surface on the Si substrate and the hydrophobicity of annealed GO samples.

## Results and Discussion

### Characteristics of GO-coated Si samples

**Figure 1:**
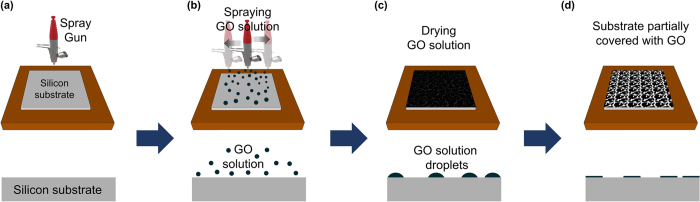
Schematic diagram for preparation of partially covered graphene oxide (GO) samples. (**a**) and (**b**) Spray coating process on Si substrate using GO-containing solution. (**c**) Drying process and (**d**) annealing process of GO-coated Si samples. In (**d**), the partially-covered GO samples on Si substrate are prepared by minimizing the size and amount of the GO-containing solution.

**Figure 2:**
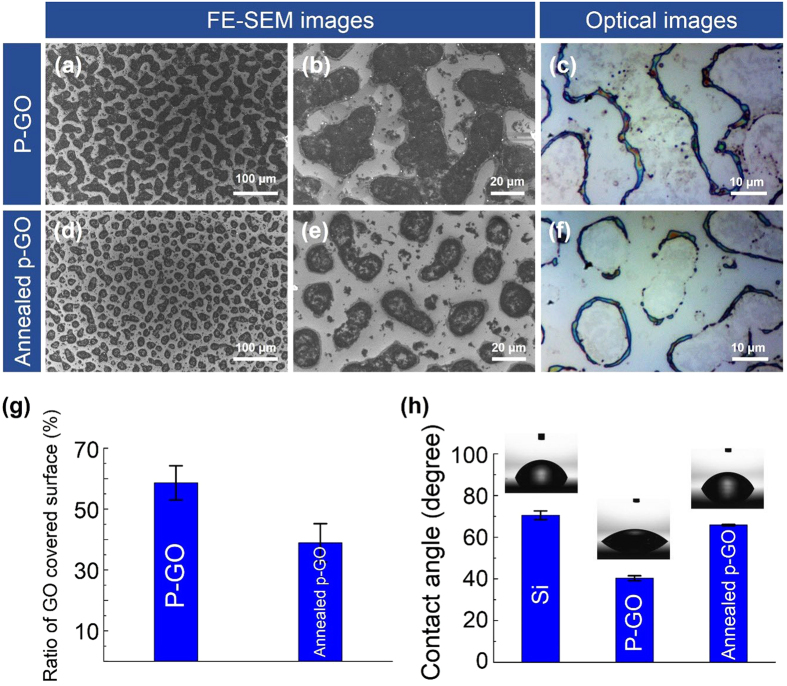
Partially GO-coated Si samples. FE-SEM images and optical images (top-view images) of (**a**–**c**) P-GO and (**d**–**f**) Annealed p-GO samples. (**g**) The ratio of GO coverage and (**h**) contact angles on the Si surface, P-GO, and Annealed p-GO samples. In (**h**), the insets show the optical images of water droplets for contact angle measurement.

**Figure 3:**
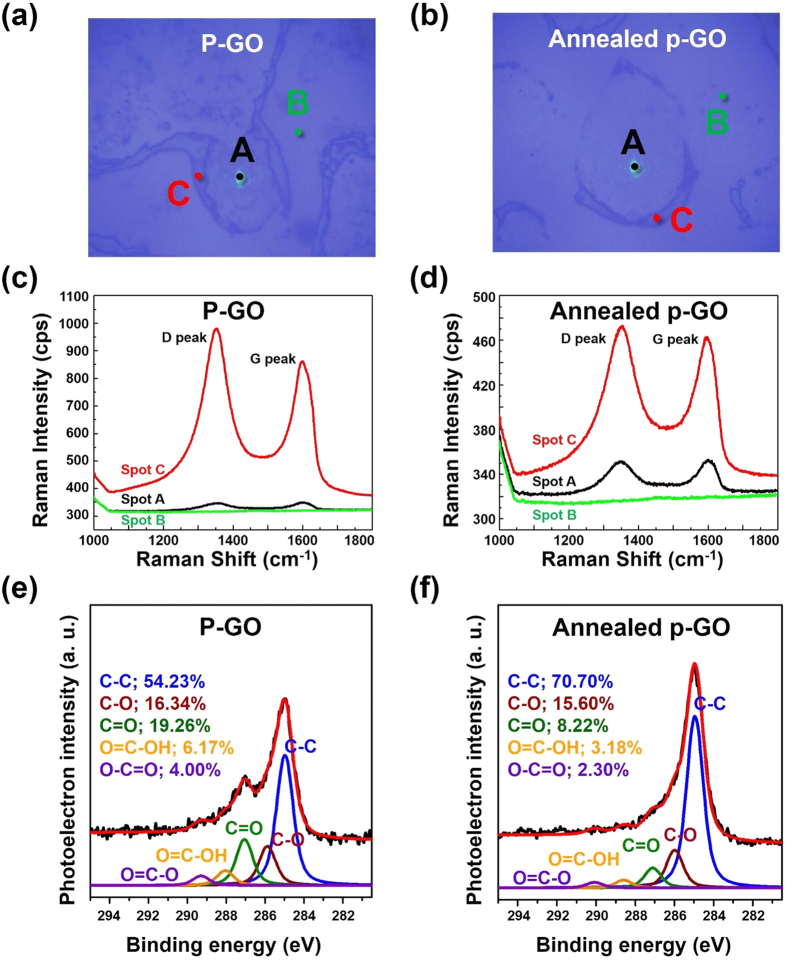
Raman and XPS measurements for partially GO-coated Si samples. (**a**,**b**) Optical images and (**c**,**d**) Raman spectra of P-GO and Annealed p-GO samples. Three different positions on partially GO-coated samples, selected as Spots A, B, and C, are positioned at the inside, outside, and boundary of the thin GO films, respectively. (**e**,**f**) C1s core-level spectra of P-GO and Annealed p-GO samples.

As shown in Fig. 1, GO-coated Si samples were prepared by the spray-coating method. On performing several repeated experiments, we found that the shape and size of GO droplets on the Si substrate were well controllable, and the conditions for spray coating were optimized (Fig. 2). In addition, by minimizing the size and amount of GO-containing solution droplets released, we were able to partially coat GO on the Si substrate as shown in Fig. 2(a–c). This method has been proven successful and has been validated several times in previous experiments. Figure 2(a–f) show field-emission scanning electron microscopy (FE-SEM) and optical images of as-prepared GO (P-GO) and annealed partially GO-coated (Annealed p-GO) Si substrates, indicating that the GO films were partially formed on the Si substrates. To control the GO occupied area on the surface of Si substrates, the GO-coated Si substrates were annealed at 900 °C in ambient H_2_ in a CVD chamber for 10 min. As shown in Fig. 2(d–f), the GO-coated area on the Si substrates was clearly reduced by ~20% compared to the P-GO samples (Fig. 2(g)). As a result, the annealing process could diminish the GO-occupied area on the Si substrate. In order to confirm the material property of GO films partially covering the Si substrates, Raman measurements were performed at room temperature. From the optical images and Raman spectra in Fig. 3(a–d), we found that round-shaped areas (Spot A and C) on both P-GO and Annealed p-GO samples have G and D peaks at ~1580 cm^−1^ and ~1350 cm^−1^, respectively, where they are normally observed in GO films^23^,^25^. As a result, the round-shaped area is confirmed to be the GO surface (Spot A and C), whereas the remaining area indicates Si surface (Spot B). Furthermore, we evaluated the GO-covered area on Si substrates by FE-SEM observation. As shown in Fig. 2(g), the GO-covered surface on the Si substrates was determined to be 58. 6 ± 5.6% and 38.9 ± 6.3% for P-GO and Annealed p-GO samples, respectively, clearly demonstrating reduced GO surface on Si substrates after the annealing process. As shown in Fig. 2(h), contact angle (CA) measurements also confirmed this observation, where the CAs were ~ 40.3°and ~ 65.8° for P-GO and Annealed p-GO samples, respectively. In comparison to a bare Si substrate (CA ~ 70.5°), the P-GO sample exhibited relatively more hydrophilic nature. On the contrary, the Annealed p-GO sample demonstrated relatively more hydrophobic property than the P-GO sample. To further investigate the effect of GO-coated topographies on wetting characteristics, we have additionally prepared fully-coated GO (F-GO) and thermally annealed fully-coated GO (Annealed f-GO) samples. The CAs for the F-GO and Annealed f-GO were determined to be approximately 27.7° and 57.2°, respectively as shown in Fig. S1(a) (see Supplementary Information (SI)). These results clearly demonstrated that the fully-coated GO samples become more hydrophilic when compared to the partially-coated GO samples.

X-ray photoelectron spectroscopy (XPS) measurements were also performed to confirm the effect of functional groups present in as-prepared P-GO and Annealed p-GO on their hydrophilicities, Furthermore, the C1s core-level spectra were deconvoluted into various chemical states using a Gaussian-Lorentzian curve fitting after performing a Shirley background correction. Figure 3(e–f) clearly show the changes in the shapes of C1s core-level spectra before and after thermal annealing process, indicating the different C-bonding configuration in P-GO and Annealed p-GO. The C1s core-level spectrum of the P-GO can be fitted to five peaks centered at 285.0, 286.0, 287.1, 288.2, and 289.4 eV, which were assigned to the sp^2^/sp^3^ (C-C/C=C, 56.89%), hydroxyl (C-O, 13.50%), carbonyl (C=O, 20.08%), carboxyl (O=C-OH, 5.62%), carboxylate (O-C=O, 3.91%) bonds^17^,^18^,^19^, respectively. For the Annealed p-GO, the intensity of the C-C/C=C peak was significantly increased, and the percent composition of this bond was estimated to be 70.70%, which is much higher than that of the P-GO (56.89%). Meanwhile, the intensities and percent compositions of other functional groups were slightly reduced as shown in Fig. 3(f). Based on our XPS results in conjunction with the wetting characteristics, as-prepared P-GO has a large amount of hydrophilic functional groups (i.e., carbonyl and hydroxyl) attached onto its basal plane or edges, which can be converted from hydrophobic to hydrophilic wetting state on Si substrate. On the contrary, the Annealed p-GO demonstrated a hydrophobic property due to the reduction of hydrophilic functional groups during thermally annealing treatment^26^,^27^.

### Analysis of cultured BT-20 cells on GO samples

**Figure 4:**
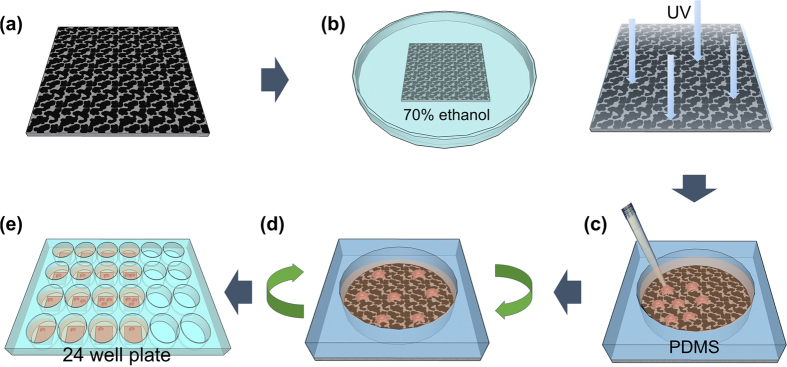
Schematic diagram of cell culture process. (**a**) Partially GO-coated Si samples (1 cm × 1 cm). (**b**) Sterilization process in 70% ethanol for 2 h and overnight UV exposure. (**c**) Cell seeding on the partially GO-coated Si samples, in which samples are covered with a PDMS well (0.8 cm in diameter). (**d**) Shaking the samples using a 3-D rocker for 3 min. (**e**) After peeling off the PDMS well, the BT-20 cells on partially GO-coated Si samples are loaded into a 24-well culture plate and incubated for 1, 12, 24, and 48 h, respectively in an incubator (37 °C, 5% CO_2_).

**Figure 5:**
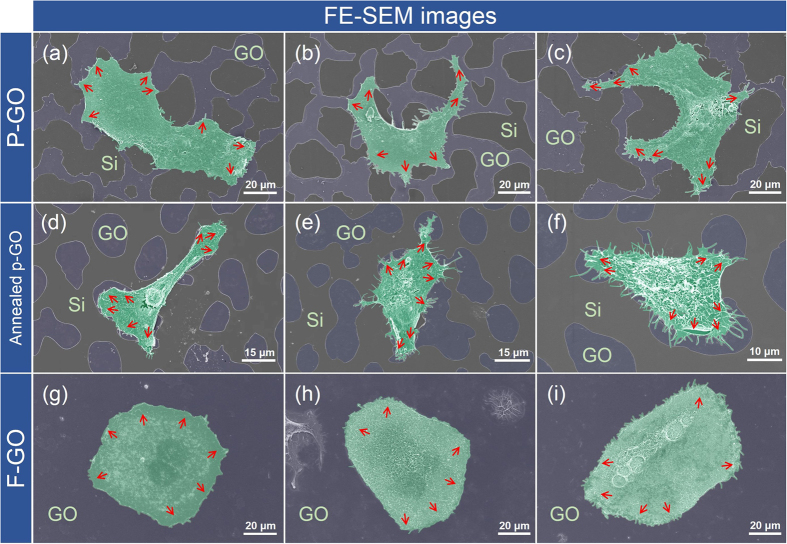
Morphology of cultured BT-20 cells on partially or fully GO-coated Si samples. FE-SEM images (top-view images) of surface-bound BT-20 cells on (**a**–**c**) P-GO, (**d**–**f**) Annealed p-GO, and (**g**–**i**) F-GO samples. The surface-bound BT-20 cells tend to spread on the GO-coated area of the partially GO-coated samples. The red-colored arrows denote the spreading direction of surface-bound BT-20 cells on partially or fully GO-coated samples.

To investigate the effects of hydrophilicity on the adhesion and growth behavior of living cancer cells, two types of GO-coated Si substrates (i.e., P-GO and Annealed p-GO samples) were mainly used for this study. We first seeded BT-20 cells (breast cancer cell line) on both the P-GO and Annealed p-GO samples and incubated them for 1, 12, 24, and 48 h in an incubator (37 °C in 5% CO_2_) (Fig. 4). We then performed FE-SEM observation to quantify the morphological properties of cultured BT-20 cells bound to both the P-GO and Annealed p-GO samples using a cell freezing technique reported previously^5^,^6^,^7^. Briefly, the samples were fixed with 4% glutaraldehyde (GA, Sigma-Aldrich, USA), followed by post-fixing in 1% osmium tetroxide, dehydration by successive immersion in 25, 50, 75, 95, and 100% ethanol, and slow drying under vacuum for 24 h. The quantitative characterizations of cellular morphologies on GO-coated Si substrates with different hydrophilicity were performed by FE-SEM imaging. Figure 5(a–f) represent the FE-SEM images of BT-20 cells bound to the surfaces of GO-coated Si substrates after 2-day culture in an incubator. These figures clearly indicate that BT-20 cells are well-formed and spread on the GO surface area, and are stretched out toward the GO surface. In contrast, cells on the Si substrate area (i.e., hydrophobic area) are hindered in initial cell adhesion, and are pushed toward the GO surface areas. Furthermore, the P-GO surface leads to enhanced cell adhesion and growth with higher adhesion force between the cells and the GO surface due to lower CA compared with the cells on the Annealed p-GO surface and Si surface as shown in Fig. 2(h) and Fig. 5 (d–f). In addition, the P-GO samples, with a lower CA of ~40.3°, exhibited a higher ratio of cell-covered area than the annealed p-GO samples (CA of ~ 65.8°). To further investigate the effect of GO-coated topographies on the adhesion and growth behavior of living cancer cells, BT-20 cells were cultured on the fully GO-coated (i.e., F-GO and Annealed f-GO) and fully graphene-coated (F-Gr) samples after 48 h culture in an incubator. As a result, the BT-20 cells are well-formed and are stretched out in all directions on the surface of fully-coated GO samples (Fig. 5(g–i) and Fig. S2(a–c) of SI). On the other hand, the BT-20 cells cultured on F-Gr samples are stretched out in restricted directions due to the deficiency of hydrophilic functional group (Figs S1(b) and S2(d,e) of SI). Similar behavior could be found on hydrophobic coated structures with various CAs in a previous report^28^. According to these results, the BT-20 cells adherent on the GO surface tend to spread out easily with a lower CA. Lee *et al*. reported that the GO surface binds with serum proteins in culture media via electrostatic interactions due to the presence of oxygenated groups^20^. Consequently, cell attachment to the GO surface is enhanced due to a higher density of surface-bound molecules compared to the control Si surface.

**Figure 6:**
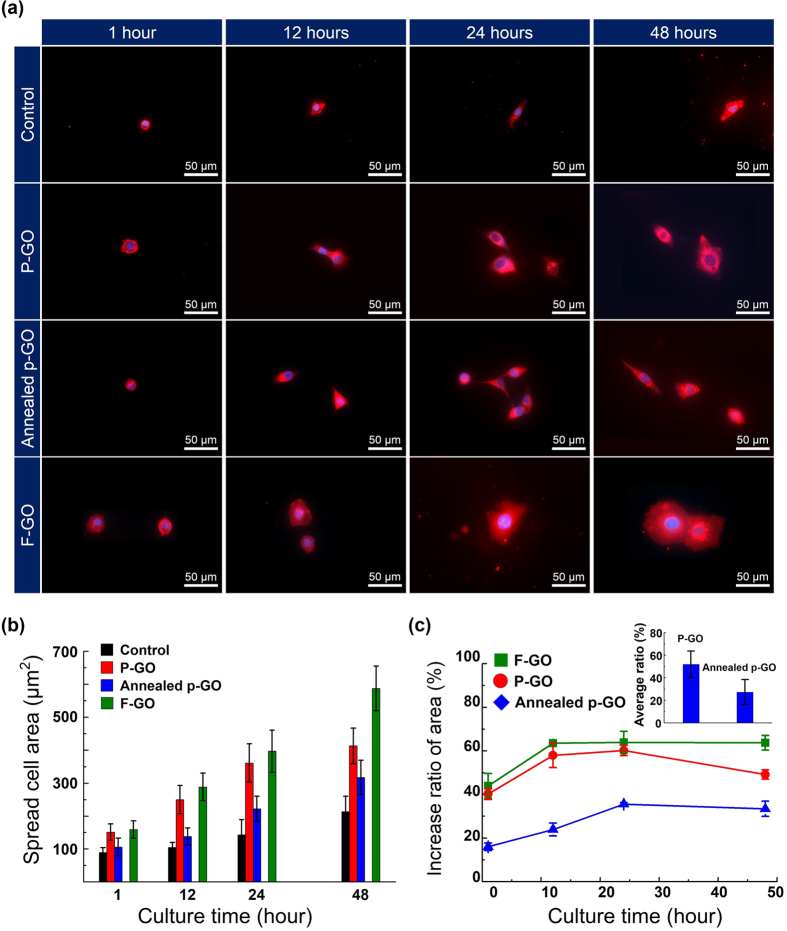
Spreading cell area and growth rate of cells on samples. (**a**) Fluorescence images of cultured BT-20 cells on P-GO, Annealed p-GO, and F-GO samples together with the control Si sample as a function of incubation time up to 48 h. The surface-bound cells are stained by both DiI (cell membrane, red) and DAPI (cell nucleus, blue) for identifying the spreading area of the cells. (**b**) The average spreading areas of the surface-bound cells on these samples as a function of the incubation time up to 48 h. (**c**) The growth rate of cells covering the P-GO, Annealed p-GO, and F-GO samples with respect to the Si sample (control). Inset of right of (**c**) shows the averaged increased ratio of cell spread area for all culture times.

To quantify cell spreading, the number of surface-bound cells, and cell morphology on two different surfaces (i.e., P-GO and Annealed p-GO samples), along with the control Si sample, we measured the fluorescence images of DiI and DAPI-stained BT-20 cells on different surfaces using a fluorescence microscope (EVOS^TM^, AMG, USA) in at least 5 areas for each sample. The acquired images were then analyzed with ImageJ software (Bethesda, MD, USA). Figure 6(a) shows overlapped fluorescence images of cultured BT-20 cells on the P-GO and Annealed p-GO samples as a function of culture time up to 2 days in an incubator, where all the cells stained with DiI (membrane in red in the figure) and DAPI (nucleus in blue in the figure) were considered for further analysis of spreading area. This figure shows that the surface-bound BT-20 cells were well-formed on both P-GO and Annealed p-GO samples. At 1 h of incubation, the BT-20 cells were immobilized on both kinds of GO surfaces with differing GO coverage on Si substrates, showing round-shaped cells on the surface and similar morphology on both substrates. With an increase in incubation time to 12 h, cells on both substrates showed slightly different morphologies, indicating that cells on the P-GO samples begin to enhance their spreading on the surface compared to the Annealed p-GO samples. With further increasing incubation time up to 48 h, it was clearly noted that the spreading area of BT-20 cells on the P-GO samples was larger than that on the Annealed p-GO samples as shown in Fig. 6(a). In Fig. 6(b), the average spreading areas of P-GO samples at the incubation times of 1, 12, 24, and 48 h were determined to be ~151, ~250, ~363, and ~413 μm^2^, respectively, whereas Annealed p-GO samples showed spread areas of ~106, ~138, ~222, and ~317 μm^2^, respectively. It indicates that the surface-bound cells on a lower CA-surface (P-GO samples) show enhanced cell spreading with increasing incubation time up to 2 days. On the other hand, the control sample (Si surface) exhibits approximately two times lower speeding areas than those for P-GO samples with an incubation time of up to 2 days (Fig. 6(b)). Figure 6(c) shows the growth rate of cells covering the P-GO and Annealed p-GO samples with respect to the control Si sample, clearly indicating that the surface-bound cells on the P-GO samples (~51%) have roughly ~24% enhancement in cell growth rate with respect to the Si samples than on the annealed GO-coated Si samples (~27%). This result can be explained by the hydrophobic property of the Annealed p-GO samples as shown in Fig. 2(h). Previous studies suggest that the hydrophobic property of surface prevents protein adsorption to the surface and affects cell adhesion on that surface^20^,^22^. Therefore, cell migration and spreading are highly restricted by the hydrophobicity of the coated surface, which can be confirmed by the quantitative analysis of the BT-20 cells cultured on the fully GO-coated (i.e., F-GO, Annealed f-GO) and F-Gr samples shown in Fig. 6 and Fig. S3(a,b) of SI. For the BT-20 cells on the F-GO and Annealed f-GO samples, the average spreading areas slightly increased when compared with those on the P-GO and Annealed p-GO samples, due to increase of their hydrophilic properties. Meanwhile, the BT-20 cells cultured on F-Gr samples exhibited smaller than those of F-GO and Annealed f-GO samples, which is responsible for the strong hydrophobic nature of F-Gr samples (CA of ~86.1°).

**Figure 7:**
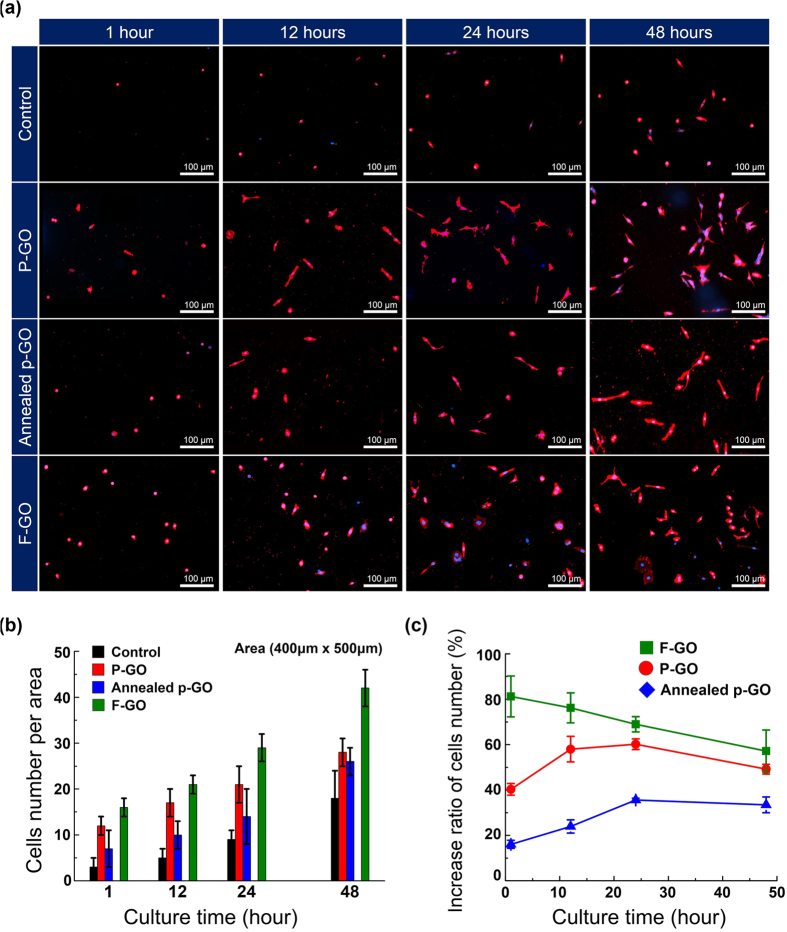
The number and growth rate of cells on the samples. (**a**) Fluorescence images of cultured BT-20 cells on P-GO, Annealed p-GO, and F-GO samples together with the control Si sample as a function of incubation time up to 48 h. (**b**) The average number of cells counted by fluorescence microscopy (EVOS^TM^, AMG, USA) in at least 5 areas (400 × 500 μm^2^) on these samples. (**c**) The increase ratio of the average number of the cells bound on P-GO, Annealed p-GO, and F-GO samples with respect to Si samples (control).

In addition, we also evaluated the number of cells per selected area (400 μm × 500 μm). As shown in Fig. 7(a,b), the average number of cells bound on P-GO samples and Si surfaces (control) was ~12, ~17, ~21, and ~28 cells after an incubation of 1, 12, 24, and 48 h, respectively. On the other hand, the number of cells on the Annealed p-GO samples was ~7, ~10, ~14, and ~26 for 1, 12, 24, and 48 h incubation, respectively, revealing that the number cells linearly increased with increasing culture time up to 48 h for all samples. Similarly, we could explain this behavior by the hydrophobic property of the Annealed p-GO samples as discussed above. Figure 7(c) shows the increase ratio of the average number of cells bound on P-GO and Annealed p-GO samples with respect to Si control samples. As shown in Fig. 7(c), it initially seems as though there is ~75% and ~57% difference in cell population, for each of the P-GO and Annealed p-GO samples, respectively, between those culture in a limited area (covered area of ~39 and ~60% with respect to Si surface, Fig. 2(g)) and those in the external areas (i.e., Si surface). However, after a maximum culture time of 48 h, the two samples yield similar results (31–35%, Fig. 7(c)). Thus, our findings here demonstrate that the cell number cultured on the GO surface initially appears to proliferate, but fails to do so over time. This is caused by the limited area of GO surface in Si substrate, which in turn decreases the cell number over time. Additionally, the number of BT-20 cells adhered on fully-coated samples (i.e., F-GO, Annealed f-GO, and F-Gr) that was strongly dependent on their hydrophobicity, as shown in Fig. 7 and Fig. S3(c,d) of SI. This study demonstrates that the hydrophobicity of GO-coated Si substrates can be easily tunable by controlling both the amount of hydrophilic functional groups and the surface topography, which is useful to study the effect of hydrophobicity on the adhesion and growth behaviors during the incubation of living cancer cells.

## Conclusion

In summary, we performed in-depth analysis of cell adhesion, growth behavior, and surface morphology on P-GO and Annealed p-GO samples, which were partially covered by the GO films on Si substrates, as a function of incubation time of up to 2 days. Analysis of cells on both GO-coated substrates revealed enhanced spreading on the as-prepared GO sample surface compared to the annealed GO samples with increase in incubation time up to 48 h. We found that the spreading area covered by the cells and the number of cells for a given culture time in an incubator were highly dependent on the hydrophobicity and the presence of oxygenated groups in GO and on the Si substrate. In this study, we demonstrated a method of fabricating partially covered GO layers on Si substrates with varying coverage of the GO layer by minimizing the size and amount of the GO-containing solution and a further annealing process. This method is a promising approach for further studies on controlling cell adhesion and growth on these surfaces.

## Methods

### Fabrication and characterization of graphene oxide (GO)-coated Si substrates

The GO solution (concentration, 0.5 g/l) was purchased from Graphene Supermarket, USA. Monolayer graphenes (Gr) were grown using by a chemical vapor deposition (CVD) method and transferred onto Si substrates. The GO-containing solution was dissolved in deionized (DI) water to obtain a concentration of 0.025 g/l. Figure 1(a–d) show the schematic diagram of the GO coating processes used on the Si substrate. The GO-containing solution was coated onto the Si substrates (1 cm × 1 cm) by the spray-coating method and the GO-coated Si samples (Fig. 1(a,b)) were then dried in air at room temperature (Fig. 1(c)). In order to compare cell adhesion and growth behavior on GO-coated Si substrates with differing hydrophobicities, we prepared annealed GO samples by an annealing process at a temperature of 900 °C in ambient H_2_ in a CVD chamber for 10 min.

Characterizations of GO- or Gr-coated Si samples were carried out by Raman spectroscopy and X-ray photoelectron spectroscopy (XPS). Raman spectroscopy (SPEX TRIAX 552, France, Renishaw) with a laser operating at a wavelength of 532 nm was used for structural characterization of the GO- or Gr-coated films. The surface chemical bonds were determined by XPS (Thermo Science, K-Alpha) using an Al Kα (hν = 1486.6 eV) X-ray source with a spot diameter of 500 μm. Additionally, the C1s spectra were deconvoluted to specific chemical states using a Gaussian-Lorentzian curve fitting after performing a Shirley background correction. The wetting characteristics of these samples were measured using by a water contact angle analyzer (Phoenix 300 plus, SEO Co., Ltd.).

### Cell culture

The schematic diagram of cell preparation on GO-coated Si substrates is shown in Fig. 4. Both as-prepared and annealed GO-coated Si samples were first sterilized with 70% ethanol for 2 h (Fig. 4(a,b)). The samples were then exposed at least overnight to an ultraviolet (UV) lamp. The polydimethylsiloxane (PDMS, 1 cm × 1 cm) mold (Fig. 4(c)) was thermally bonded to the sterilized GO-coated Si samples after cleaning with 75% alcohol in an ultrasonic bath. A cell suspension containing ~1 × 10^3^ cells in 200 μl was seeded into the PDMS well (~8 mm in diameter) with culture media (RPMI 1640, Thermo Fisher Scientific, USA). The seeded cells in the PDMS well with the GO-coated Si substrate were evenly shaken by a 3-D rocker for 3 min (Fig. 4(d)). After peeling off the PDMS wells from the GO-coated Si substrates, the samples were placed in a 24-well culture plate. The seeded BT-20 cells on both as-prepared and annealed GO-coated Si samples were incubated for 1, 12, 24, and 48 h at 37 °C in a humidified 5% CO_2_ incubator.

### Analysis of cultured BT-20 cells on GO-coated Si samples

For this experiment, cultured BT-20 cells on GO-coated Si substrates were first stained with DiI (Vybrant cell-labeling solution; 565 nm; Thermo Fisher Scientific, USA) and DAPI (4′,6-diamidino-2-phenylindole; nuclear staining; 470 nm; Thermo Fisher Scientific, USA) to quantify the average spread area and the number of cells on the GO-coated Si samples. The samples were rinsed at least thrice with phosphate-buffered saline (PBS, ×1, Invitrogen, USA) to remove the culture media (RPMI 1640) and were stained with 1% DiI solution in PBS at 37 °C in an incubator for 20 min, followed by washing with PBS. DiI and DAPI-stained BT-20 cells on the GO-coated samples were observed using a fluorescence microscope (EVOS^TM^, AMG, USA) in at least 5 fields (400 × 500 μm^2^) for each sample. The images acquired from the fluorescence microscope were analyzed to quantify the spreading area and the number of cells on a specific area of GO-coated Si samples using ImageJ software (Bethesda, MD, USA). In addition, BT-20 cells on GO-coated Si samples were stained using both phalloidin (F-actin staining, 565 nm, Thermo Fisher Scientific, USA) and DAPI (nucleus staining, 470 nm) for confirming cell shape.

## Additional Information

**How to cite this article**: Jeong, J.-T. *et al*. Effect of graphene oxide ratio on the cell adhesion and growth behavior on a graphene oxide-coated silicon substrate. *Sci. Rep.* **6**, 33835; doi: 10.1038/srep33835 (2016).

## Supplementary Material

Supplementary Information
