# Supplementary material for: Effect of graphene oxide ratio on the cell adhesion and growth behavior on a graphene oxide-coated silicon substrate
Source: Sci Rep. 2016 Sep 22;6:33835. doi: 10.1038/srep33835 (PMC5031981; doi:10.1038/srep33835)
Supplement: Supplementary Information [file srep33835-s1.pdf]

Supplementary Information

Effect of graphene oxide ratio on the cell adhesion and growth behavior on a graphene oxide-coated silicon substrate

Jin-Tak Jeong[1], Mun-Ki Choi[1], Yumin Sim[1], Jung-Taek Lim[1], Gil-Sung Kim[1], Maeng-Je Seong[1], Jung-Hwan Hyung[2], Keun Soo Kim[3], Ahmad Umar[*4,5], and Sang-Kwon Lee[*,1]

[1]Department of Physics, Chung-Ang University, Seoul 156-756, Republic of Korea
[2]Department of Semiconductor Science and Technology, Chonbuk National University, Jeonju 561-756, Republic of Korea
[3]Department of Physics and Graphene Research Institute, Sejong University, Seoul 143-747, South Korea
[4]Department of Chemistry, College of Science and Arts, Najran University, Najran-11001, Kingdom of Saudi Arabia
[5]Promising Centre for Sensors and Electronic Devices (PCSED), Najran University, Najran-11001, Kingdom of Saudi Arabia

Supplementary Figure S1.

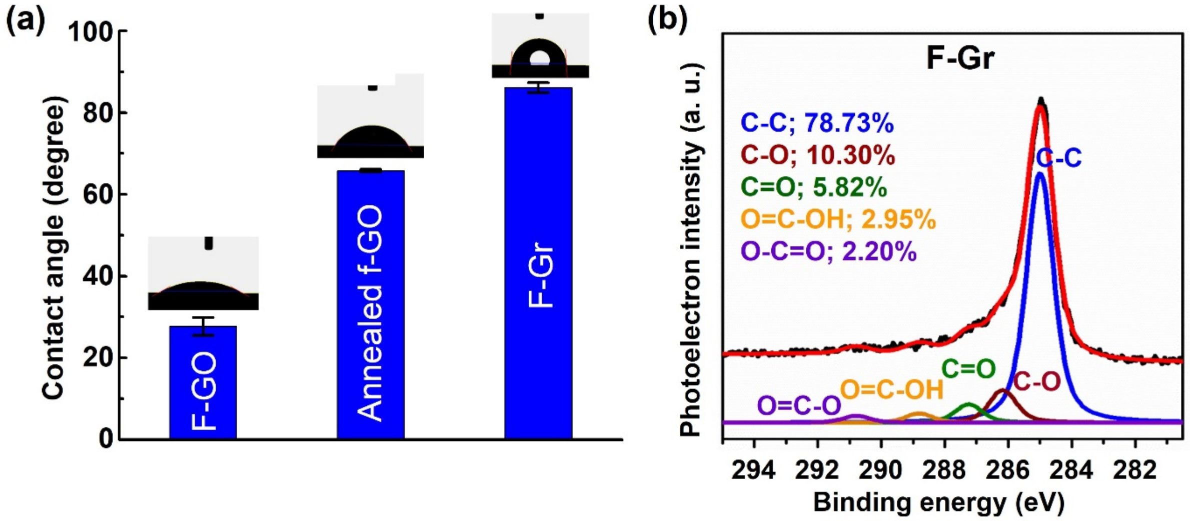

Figure S1. Contact angles and XPS measurement of fully GO-coated and fully Gr-coated samples. (a) Contact angles on the fully GO-coated (F-GO), annealed fully GO-coated (Annealed f-GO), and fully Gr-coated (F-Gr) samples. (b) XPS spectra of the F-Gr sample. In (a), the insets show the optical images of water droplets for contact angle measurement.

Supplementary Figure S2.

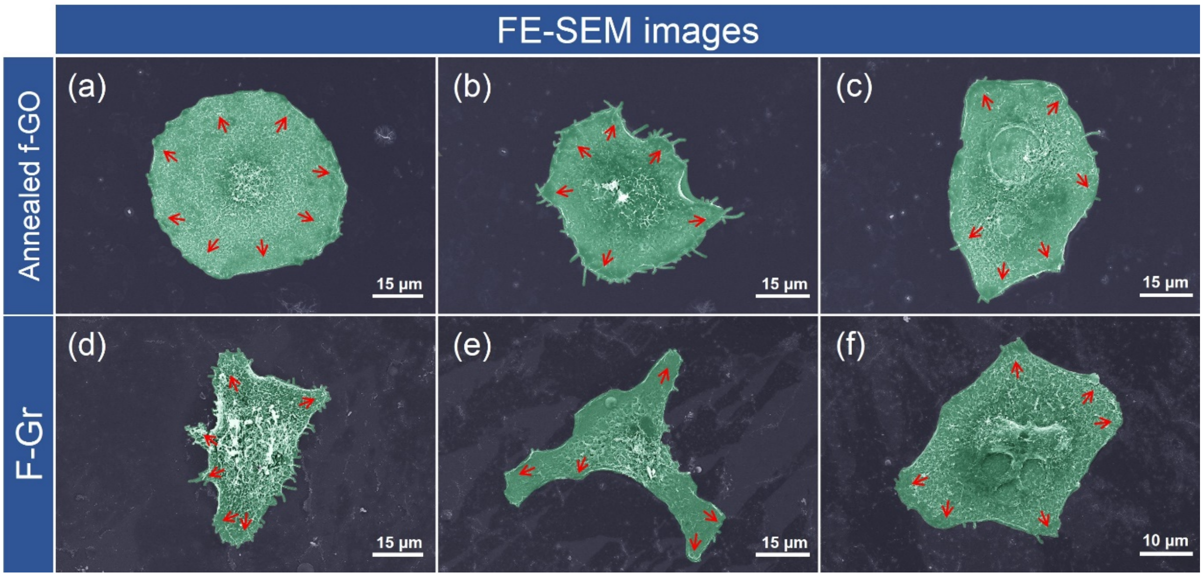

Figure S2. Morphology of cultured BT-20 cells on the Annealed f-GO and F-Gr samples. FE-SEM images (top-view images) of surface-bound BT-20 cells on (a)-(c) Annealed f-GO and (d)-(f) F-Gr samples. The red-colored arrows denote the spreading direction of surface-bound BT-20 cells.

Supplementary Figure S3.

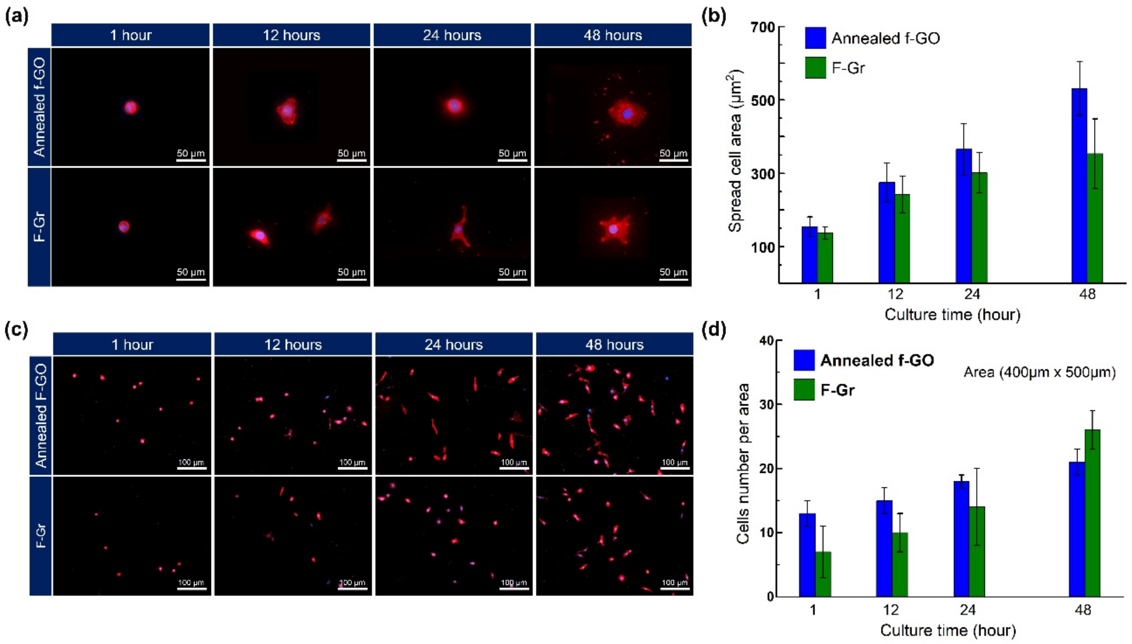

Figure S3. Spreading cell area and the number of cells on the Annealed f-GO and F-Gr samples. (a) and (c) The fluorescence images of cultured BT-20 cells on Annealed f-GO and F-Gr samples as a function of incubation time up to 48 h. The surface-bound cells are stained by both DiI (cell membrane, red) and DAPI (cell nucleus, blue) for identifying the spreading area and proliferating number of the cells. (b) The average spreading areas of Annealed f-GO and F-Gr samples as a function of the incubation time up to 48 h. (d) The average number of cells counted by fluorescence microscopy (EVOS^{TM}, AMG, USA) in at least 5 areas (400 × 500 μm^2) for Annealed f-GO and F-Gr samples as a function of incubation times up to 48 h.
